# Preparing for a Second Attack: A Lesion Simulation Study on Network Resilience After Stroke

**DOI:** 10.1161/STROKEAHA.121.037372

**Published:** 2022-05-09

**Authors:** Mitsouko van Assche, Julian Klug, Elisabeth Dirren, Jonas Richiardi, Emmanuel Carrera

**Affiliations:** Stroke Research Group, Department of Clinical Neurosciences, University Hospital and Faculty of Medicine, Geneva, Switzerland (M.v.A., J.K., E.D., E.C.).; Department of Radiology, Lausanne University Hospital and University of Lausanne, Switzerland (J.R.).

**Keywords:** connectivity, graph, magnetic resonance imaging, motor cortex, recovery, resilience, stroke

## Abstract

Does the brain become more resilient after a first stroke to reduce the consequences of a new lesion? Although recurrent strokes are a major clinical issue, whether and how the brain prepares for a second attack is unknown. This is due to the difficulties to obtain an appropriate dataset of stroke patients with comparable lesions, imaged at the same interval after onset. Furthermore, timing of the recurrent event remains unpredictable.

**Methods:**

Here, we used a novel clinical lesion simulation approach to test the hypothesis that resilience in brain networks increases during stroke recovery. Sixteen highly selected patients with a lesion restricted to the primary motor cortex were recruited. At 3 time points of the index event (10 days, 3 weeks, 3 months), we mimicked recurrent infarcts by deletion of nodes in brain networks (resting-state functional magnetic resonance imaging). Graph measures were applied to determine resilience (global efficiency after attack) and wiring cost (mean degree) of the network.

**Results:**

At 10 days and 3 weeks after stroke, resilience was similar in patients and controls. However, at 3 months, although motor function had fully recovered, resilience to clinically representative simulated lesions was higher compared to controls (cortical lesion *P*=0.012; subcortical: *P*=0.009; cortico-subcortical: *P*=0.009). Similar results were found after random (*P*=0.012) and targeted (*P*=0.015) attacks.

**Conclusions:**

Our results suggest that, in this highly selected cohort of patients with lesions restricted to the primary motor cortex, brain networks reconfigure to increase resilience to future insults. Lesion simulation is an innovative approach, which may have major implications for stroke therapy. Individualized neuromodulation strategies could be developed to foster resilient network reconfigurations after a first stroke to limit the consequences of future attacks.

**See related article, p 2048**

Does the brain become more resilient to further events, following a first stroke? Despite major progress in secondary prevention, recurrent strokes are frequent, occurring in up to 20% of patients within 3 months of onset.^1^ In stroke animal models, the behavioral impact of a second lesion decreases with time,^2^ suggesting that resilience to new events—defined here as the capacity of the brain to resist, overcome, or thrive in the face of adversity^3^—progressively builds up during recovery. In humans, it is still debated whether the occurrence of an ischemic event limits the consequences of a second event.^4–6^ If proven to be true, the hypothesis that resilience increases within days or months after stroke to circumvent the impact of a potential recurrent stroke may lead to major physiological and clinical implications.^3^ Understanding how and why the brain reorganizes in a certain configuration after stroke could help promote neuromodulatory therapeutic strategies that will aim not only at restoring function but also at promoting network configuration that minimizes the effects of a potential recurrent stroke. Investigating resilience after a first stroke in humans is, however, challenging. This is due to the difficulties to obtain an appropriate dataset of patients with comparable lesions, imaged at the same interval after stroke onset. Furthermore, timing and localization of the recurrent event remain unpredictable in a given patient.

To circumvent our inability to predict a new event, we evaluated resilience in brain networks by simulating recurrent lesions through node deletions in a population of patients with similar infarcts restricted to the primary motor cortex. In previous studies, node deletion has been key to determine that brain networks of healthy subjects are organized to optimize the balance between integration and segregation.^7–10^ By deleting nodes randomly or according to their importance in the network, it was also possible to demonstrate that brain networks architecture confers robustness despite vulnerability of central nodes.^11,12^ Deletion of contiguous nodes was only recently considered as a method to represent strokes with their anatomic characteristics, in terms of size and location.^13,14^ In humans, this method seems a particularly promising alternative to empirical studies to study resilience after stroke, given the unpredictable nature of the second clinical event.

Here, we tested the hypothesis that resilience in brain networks increases during stroke recovery. For that purpose, we considered resilience as the network capacity to maintain information capacity after a second attack, based on the measure of the global efficiency (E_glob_). Resilience was investigated by simulating 2 types of attacks. In one classical approach, nodes of whole-brain networks were serially deleted, randomly or based on their importance in the network. We then simulated clinically representative lesions and evaluated their impact on network reorganization. Operationally, we used lesion simulation in a population of stroke patients with a lesion restricted to the primary motor cortex and contralateral hand paresis at 3 time points (TPs) within 3 months of onset. All patients had a detailed motor examination and functional connectivity analyses (resting-state functional magnetic resonance imaging and graph measures) at each TP.

## Methods

The data that support the findings of this study are available from the corresponding author upon reasonable request.

### Participants

We included 16 consecutive stroke patients (6 women; age 73±12 years) with a small lesion restricted to the primary motor cortex and isolated contralateral hand paresis. These patients were prospectively recruited out of the 1656 patients admitted to our stroke center during the study period. Exclusion criteria were (1) left-handedness, (2) significant carotid or intracranial artery stenosis (>50%), (3) history of stroke or psychiatric disease. Sixteen healthy subjects, matched for age, gender, and cardiovascular risk factors (6 women; 70±10 years) were included. This cohort was used in a recent study with the distinct aim of investigating surrogates of motor recovery focusing on the peri-infarct within 3 weeks after stroke.^15^ This previous study did not include graph analysis in whole-brain networks, nor lesion simulation. Detailed measures of motor function and imaging data were obtained on the same day at 3 TPs in patients: TP1: <10 days; TP2: 3 weeks; and TP3: 3 months poststroke and at 1 TPs in healthy subjects. Consent was obtained according to the Declaration of Helsinki. The study was approved by the Geneva Ethical Committee.

### Behavior Assessment

Hand motor function was evaluated by measuring hand dexterity (9-hole pegboard task) and isometric grip strength (JAMAR dynamometer, Asimow Engineering, Co, Los Angeles, CA). A 2-point discrimination task applied to the index fingers was used to exclude sensory deficits. For subsequent analysis of dexterity and grip strength, performance of the paretic hand was normalized by the one of the nonparetic hand (paretic hand/unaffected hand). Owing to the non-normality of the data, Wilcoxon tests were used to examine changes in hand motor function.

### Imaging Acquisition

Every patient was scanned 3 times, whereas healthy subjects were scanned once. Images were acquired on a 3T magnetic resonance imaging (MRI; MAGNETOM Prisma, Siemens Healthcare, Erlangen, Germany; 64-channel head-coil) the same day as behavioral testing. Acquisition of resting-state functional images was performed using a gradient echo planar imaging sequence (echo time/repetition time=30/1200 ms, voxel size=3 mm isotropic, 400 volumes, total acquisition time 8 minutes). Continuous eye-tracking was used to check wakefulness. Respiratory movements were recorded using a transducer at the level of maximum respiratory expansion (BioPac Inc, Santa Barbara). T1-weighted anatomic scans were acquired with an MPRAGE (Magnetization Prepared - Rapid Gradient Echo) sequence (echo time/repetition time=2.27/2300 ms, voxel size=1.0 mm isotropic), together with T2-weighted (echo time/repetition time=108/6090 ms, voxel size=0.4×0.4×4.0 mm) and DWI images (echo time/repetition time: 52/4300 ms, voxel size=1.4 × 1.4 × 4.0 mm). Finally, the protocol included brain MR angiography (time of flight) and precerebral Doppler ultrasound to rule out intracranial or precerebral stenosis.

### Imaging Data Analysis

#### Imaging Data Preprocessing

Data were preprocessed using SPM12 and in-house MATLAB scripts according to an established pipeline (https://miplab.epfl.ch/index.php/software/wFC) with additional signal cleaning.^16^ First, functional images were realigned for each subject. Then, anatomic T1 images were coregistered to the mean functional image of the corresponding subject and segmented into gray matter, white matter, and cerebrospinal fluid maps. We used the Brainnetome atlas, which provides a parcellation of the human brain in 246 regions and includes a fine-grained parcellation of the motor cortex, to atlas the gray matter of each subject in native space.^17^ The resulting map was coregistered to the mean image of the functional data of the corresponding participant.

#### Extraction of Brain Signals

The first 5 volumes were discarded to account for magnetization equilibrium. Time courses were linearly detrended at each voxel, averaged for each region of the atlas, and scaled by the signal mean of the given region. The 6 motion parameters, their first derivatives, and the average signal of cerebrospinal fluid were regressed out. Additionally, respiratory movements were corrected using RETROICOR. To correct for remaining outlying spikes, time courses were winsorized to the fifth and 95th percentiles. They were then filtered into 4 frequency sub-bands using a wavelet transform (cubic orthogonal B-spline). We focus here on scale 4 of this decomposition (frequency range 0.03<*f*<0.06 Hz). We further checked for undesirable motion effects by computing the mean framewise displacements for all subjects and TPs.^18^ There was no difference in motion across TPs (Friedman test; χ^2^[2]=0.462, *P*=0.794), and no volumes were removed. Connectivity matrices were derived by computing pairwise Pearson correlation coefficients between the 246 regions of the Brainnetome atlas. Six regions of interest, for which signal drop-out was observed in at least one subject, were removed, yielding a total of 240 regions of interest. Finally, we flipped left and right hemisphere regions of interests data within the connectivity matrices level for patients with right lesions (n=4).

#### Graph Construction

**Figure 1.**
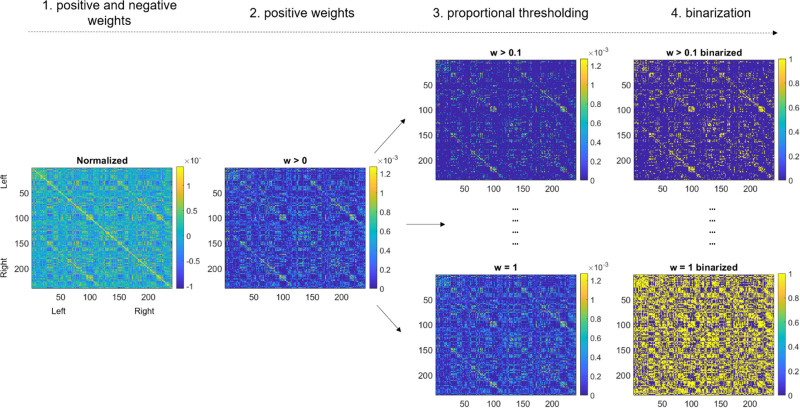
**Pipeline of graph construction.** (1) Fully connected graph containing positive and negative connectivity weights. (2) Application of an absolute thresholding w>0 to retain positive weights only. (3) Sparsely to densely connected graphs are obtained by means of proportional thresholding by step of incremental steps of 0.1. (4) Binarization of connectivity matrices leads to unweighted graphs.

Graphs were constructed following 4 steps. First, each connectivity matrix was normalized by its total connectivity strength, and this full graph was used to calculate global connectivity strength. In the next step, each connectivity matrix was thresholded using an absolute threshold *w*>0 to remove negative weights (Figure 1). A proportional (edge density) thresholding *t* was then applied, from *t*=0 (no connection) to *t*=1 (all connections retained) with a density increment of 0.1. This procedure allows filtering connectivity weights according to the strongest weights in a cumulative manner. Thus, this approach precludes the use of an arbitrary threshold and allows examining graph properties over a range of edge density values instead. Finally, each matrix was binarized before computing all other graph metrics. To derive efficiency and cost in the network, we used the brain connectivity toolbox.^19^

#### Graph Metrics

The whole network efficiency was estimated using the measure of global efficiency (*E*_*glob*_).^8,11,20^ This metric provides a measure of information transfer across all nodes of the network. It quantifies the extent to which nodes communicate with distant nodes. It is proportional to the inverse of the shortest path length.^21^


$$E_{glob}=\frac{1}{N}\sum_{i\in V}E_{i}=\frac{1}{N}\sum_{i\in V}\frac{\sum_{j\in Vj\neq id_{ij}^{-1}}}{V-1}$$

where E_i_ is the efficiency of node *i*, *V* is the set of all nodes in the network, and *N* is the number of nodes. (*i*, *j*) is a link between nodes *i* and *j*, (*i*, *j ∈ V*), *d*_ij_ is the shortest path length (distance) between *i* and *j*.

As global efficiency depends on network density, we checked the density range in which graphs remained connected in each subject. As a result, we retained the density range 0.3 to 1 for subsequent analyses rather than selecting arbitrary thresholds. We then derived the area under the curve (AUC) over the selected density range for E_glob_:


$$\text{AUC}\left(E_{glob}\right)=\int_{0.3}^{1}E_{glob}(\delta )$$

The resilience of the network was determined as the measure of network efficiency (*E*_*glob*_) after lesion simulation.

The total wiring cost or density of the network was estimated using the mean node degree of the network which can be estimated as the number of edges connected to each node, averaged over all nodes of the network. AUC(mean degree) was computed in the same fashion as AUC(E_glob_)

For better readability of the manuscript, we will use the terms E_glob_ and mean degree instead of AUC(E_glob_) and AUC(mean degree) in the next sections.

### Statistical Analysis

#### General Strategy

To investigate changes in resilience after stroke, we compared global efficiency (E_glob_) in whole-brain functional networks at 3 TPs within 3 months of stroke in 16 patients with a lesion restricted to the primary motor network. We evaluated the impact of different simulated lesions (node deletion), beginning with random and targeted attacks in the whole-brain network and then using attacks mimicking cortical and subcortical strokes that can be observed in clinical practice. For both the spontaneous evolution and the impact of simulated attack, we first used a linear mixed model to capture the global evolution along time points. *T* tests were then used to compare measurements between individual TPs and between patients and controls. Finally, we evaluated whether changes in resilience were correlated with changes in total wiring costs of the network.

##### Spontaneous Changes in Global Network Efficiency and Mean Degree During Recovery

E_glob_ and mean degree were measured at each of the 3 TPs in patients (10 days, 3 weeks, 3 months) and in controls. To evaluate changes in global efficiency over time in patients, we first used a linear mixed model with E_glob_ as the dependent variable, TP, lesion volume, and lesion side as fixed effects and subjects as a random effect. Significance was evaluated by using the Satterthwaite approximations for *df*.^22^ Within patients, longitudinal comparisons between E_glob_ at the 3 TPs were then performed using paired *t* tests with false discovery rate corrections (Benjamini-Hochberg procedure) for multiple comparisons.^23^ Between patients and controls, comparisons between the E_glob_ for patients at each TP and the E_glob_ for controls at their single TP were performed by a *t* test, with false discovery rate correction. Mean degree was analyzed analogously.

##### Impact of Lesion Simulation on Resilience in Brain Networks

###### Random and Targeted Attacks.

We first deleted nodes in random order. Global efficiency was recalculated after each node deletion (ie, 1–240 node deletions) and then averaged at each density threshold (30%–100%) for each patient. AUC were then derived as described above, resulting in one value per patient at each TP and in one single value per control. We then performed a similar analysis with nodes deleted based on their importance within the network (targeted attack, ie, by decreasing order of node degree). For comparison between TPs in patients and between patients and healthy controls, we first used a linear mixed model using the same fixed and random effects as described above followed by *t* tests with false discovery rate correction for multiple comparisons.

###### Clinically Representative Attacks.

**Figure 2.**
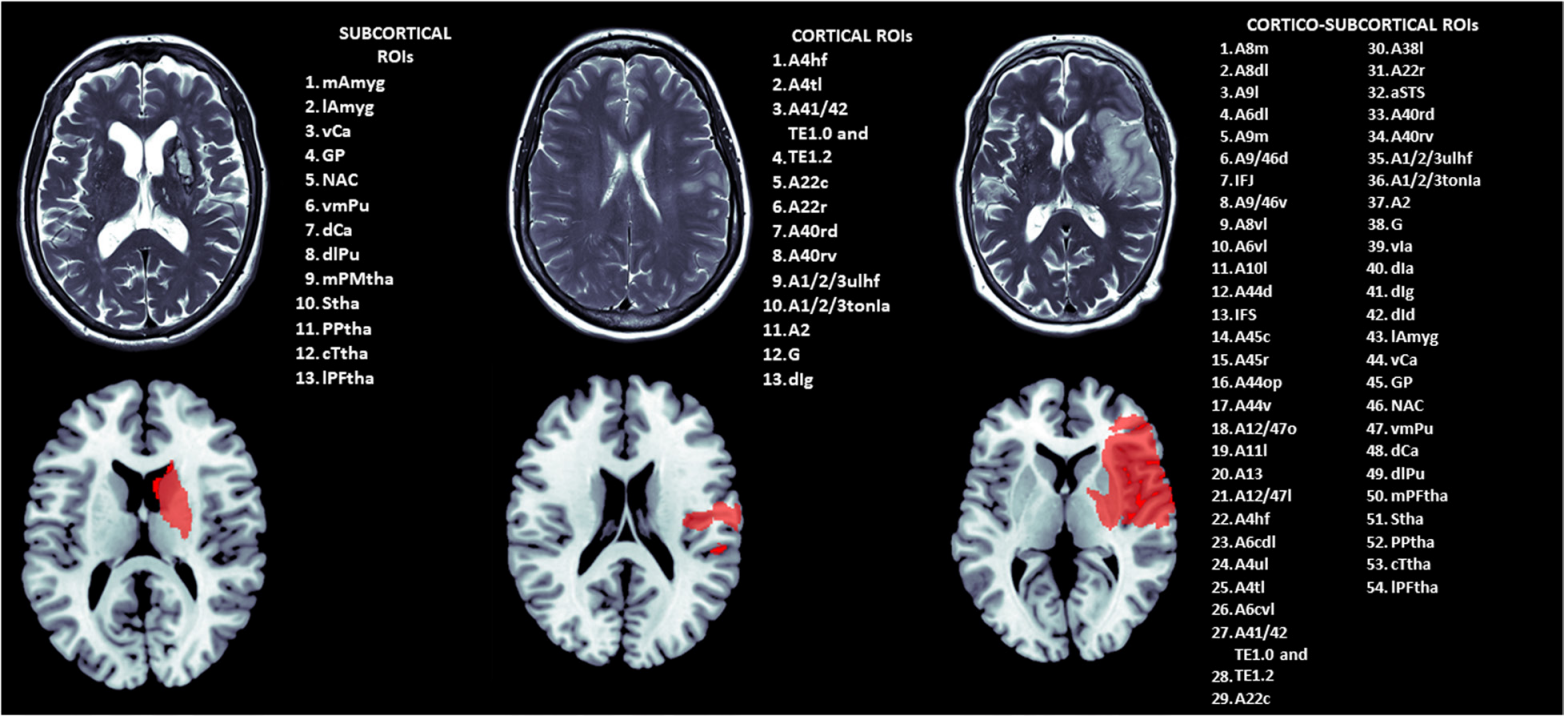
**Lesion masks of focal middle cerebral artery (MCA) strokes used for lesion simulation with corresponding Brainnetome regions. Top**, Magnetic resonance imaging (T2 sequence) shows the lesion for each stroke subtype (subcortical, cortical, cortico-subcortical MCA territory). Bottom: standard Montreal Neurological Institute template with an overlay of the lesion mask (in red) resulting from the delineation of the infarct shown in the **top** row after normalization to the template. The list of regions of interest (ROIs) enumerates the Brainnetome atlas regions overlapping with the lesion masks. A detailed description of Brainnetome ROIs can be found in Table S3.

We mimicked 3 typical representative strokes (cortico-subcortical, subcortical, and cortical lesions; Figure 2) by deleting nodes included in lesion masks corresponding to lesions of 3 patients admitted to our stroke center.^24,25^ We chose infarction in the territory of the middle cerebral artery (MCA) because it is the most frequently affected by ischemic strokes. The 3 lesion masks were manually outlined on the T2 MRI and the resulting masks normalized to Montreal Neurological Institute space with the Clinical Toolbox.^26^ As a result, the subcortical and cortical masks included 13 nodes each (respective volumes: 13.3 cm^3^ and 10.2 cm^3^), and the cortico-subcortical mask comprised 54 nodes (volume: 99.0 cm^3^). E_glob_ was computed after node deletion and the AUC was computed over the density spectrum as described above. For comparison between TPs in patients and between patients at each time point, we first used a linear mixed model using time points, attack type, lesion volume, and lesion side as fixed effects with an interaction term between time points and attack type, and subjects, as a random effect. We then performed *t* tests to compare AUC between TPs and healthy controls using false discovery rate corrections for multiple comparisons as described above.

##### Correlation Between Wiring Cost of Resilience of the Networks

To determine the price of changes in resilience between time points 2 and 3, we correlated the changes in resilience (E_glob_) and the changes in total wiring cost (mean degree).

## Results

### Changes in Motor Behavior

Hand dexterity improved from TP1 to TP2 (9-hole peg test; median laterality ratio at TP1: 1.18 (interquartile range, 1.06–1.71); at TP2: 1.03 (interquartile range, 0.94–1.27) *P*=0.01). At TP2, patients had fully recovered with no differences from healthy controls (median laterality ratio in controls=1.06 [interquartile range, 0.98–1.1]; *P*=0.98). There was no change in hand dexterity between TP2 and TP3 (*P*=1.0). Grip strength remained stable over time (JAMAR dynamometer; TP1–TP2: *P*=0.41; TP2–TP3: *P*=0.06) and was not different from controls at any TP (TP1: *P*=0.14; TP2: *P*=0.21; TP3: *P*=0.38).

#### Changes in Global Efficiency and Mean Degree During Recovery

**Figure 3.**
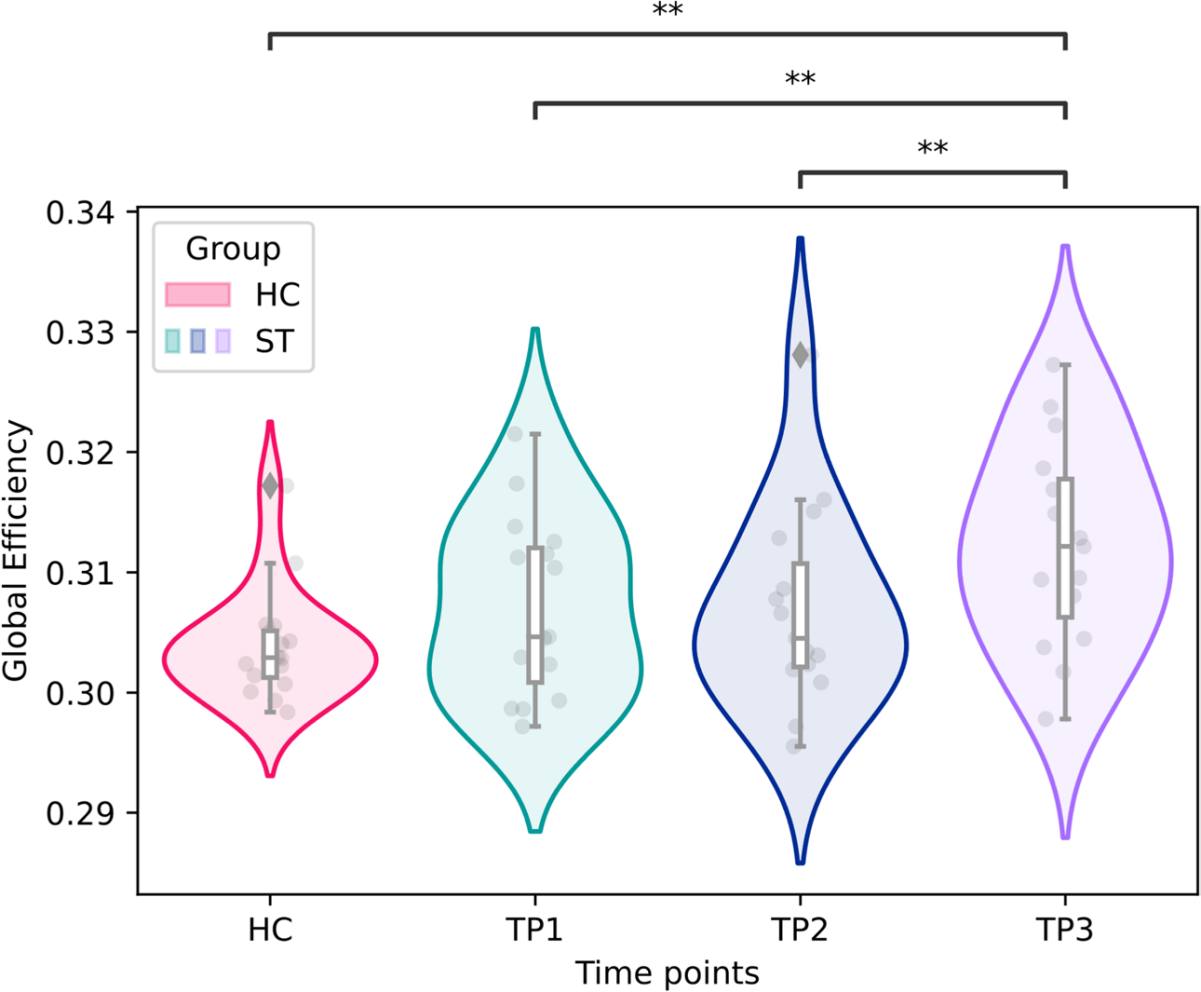
**Changes in network efficiency over time.** Violin plots with inner box plots for areas under the curve of global efficiency in stroke patients (ST, shown in green, blue, and purple) and healthy controls (HC, shown in red) at each time point (TP). Each box extends from the 25th percentile to the 75th percentile with a line indicating the median. Upper and lower whiskers show the range up to the upper and lower extremes (±1.5×interquartile range). Individual values are represented by gray dots. Outliers are represented by gray diamond shapes. Significance was evaluated with multiple *t* tests with false discovery rate corrections; *P* values are represented as *P≤0.05 and **P≤0.001.

**Figure 4.**
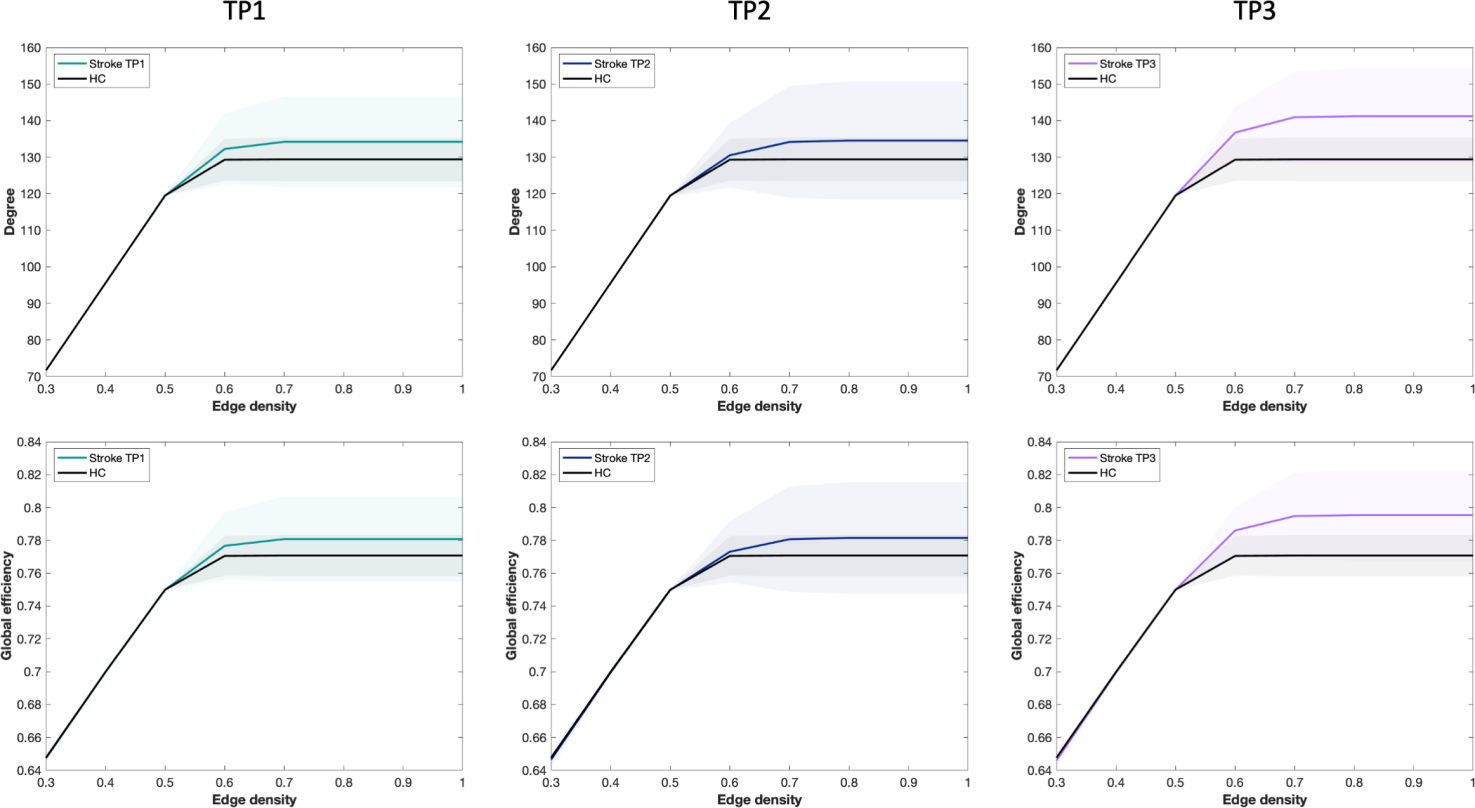
**Comparison of network cost (degree) and global efficiency between patients and healthy controls at different time points (TP).** Representation of mean degree and global efficiency illustrated with means (lines) and standard deviations (filled areas) in patients and healthy controls (HC) at each TP.

There was a statistically significant difference in E_glob_ between TPs (mixed model, F=8.534; *P*=0.007). E_glob_ was similar between TP1 to TP2 (*P*_BH_=0.941) and between patients and controls at TP1 (*P*_BH_=0.222) and TP2 (*P*_BH_=0.222). However, E_glob_ increased from TP2 to TP3 (*P*_BH_=0.017) and was higher in patients at TP3 compared to controls (*P*_BH_=0.006; Figures 3 and 4).

Mean degree increased significantly during recovery (mixed model, F=5.406, *P*=0.028). Mean degree was similar between TP1 to TP2 (*P*_BH_=0.934) and between patients and controls at TP1 (p_BH_=0.246) and TP2 (*P*_BH_=0.246). However, E_glob_ increased from TP2 to TP3 (*P*_BH_=0.030) and was higher in patients at TP3 compared to controls (*P*_BH_=0.009; Figure S1).

#### Impact of Lesion Simulations on Network Resilience

##### Random Failure

**Figure 5.**
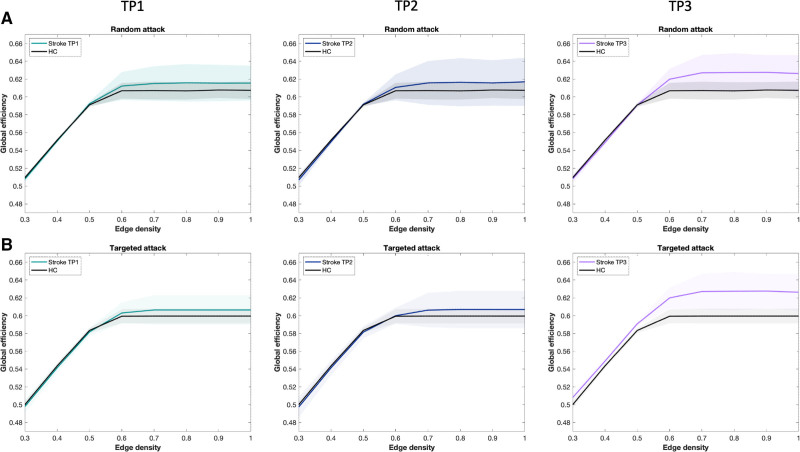
**Network resilience after serial random and targeted attacks.** Representation of global efficiency after (**A**) random node deletion and (**B**) targeted node deletion based on degree. This representation is illustrated with mean global efficiency (lines) and SDs (filled areas) after attacks for patients at time point (TP) 1, TP2, and TP3, as well as for healthy controls (HC).

There were significant changes in E_glob_ over time after random attacks (mixed model, F=5.360, *P*=0.028). E_glob_ did not differ after random attacks between patients and controls at TP1 (*P*_BH_=0.279) nor TP2 (*P*_BH_=0.279). However, patients displayed higher E_glob_ at TP3 compared to controls (*P*_BH_=0.012) and TP2 (*P*_BH_=0.012; Figure 5).

##### Targeted Attack

Using the measure of degree to target serially the nodes of the network, E_glob_ differed from control only at TP3 (*P*_BH_=0.015). Longitudinally, E_glob_ significantly varied across TPs (mixed model, F=7.954, *P*=0.009). E_glob_ increased from TP2 to TP3 (*P*_BH_ =0.015) but not between TP1 and TP2 (*P*_BH_=0.846).

##### Clinically Representative Lesions

**Figure 6.**
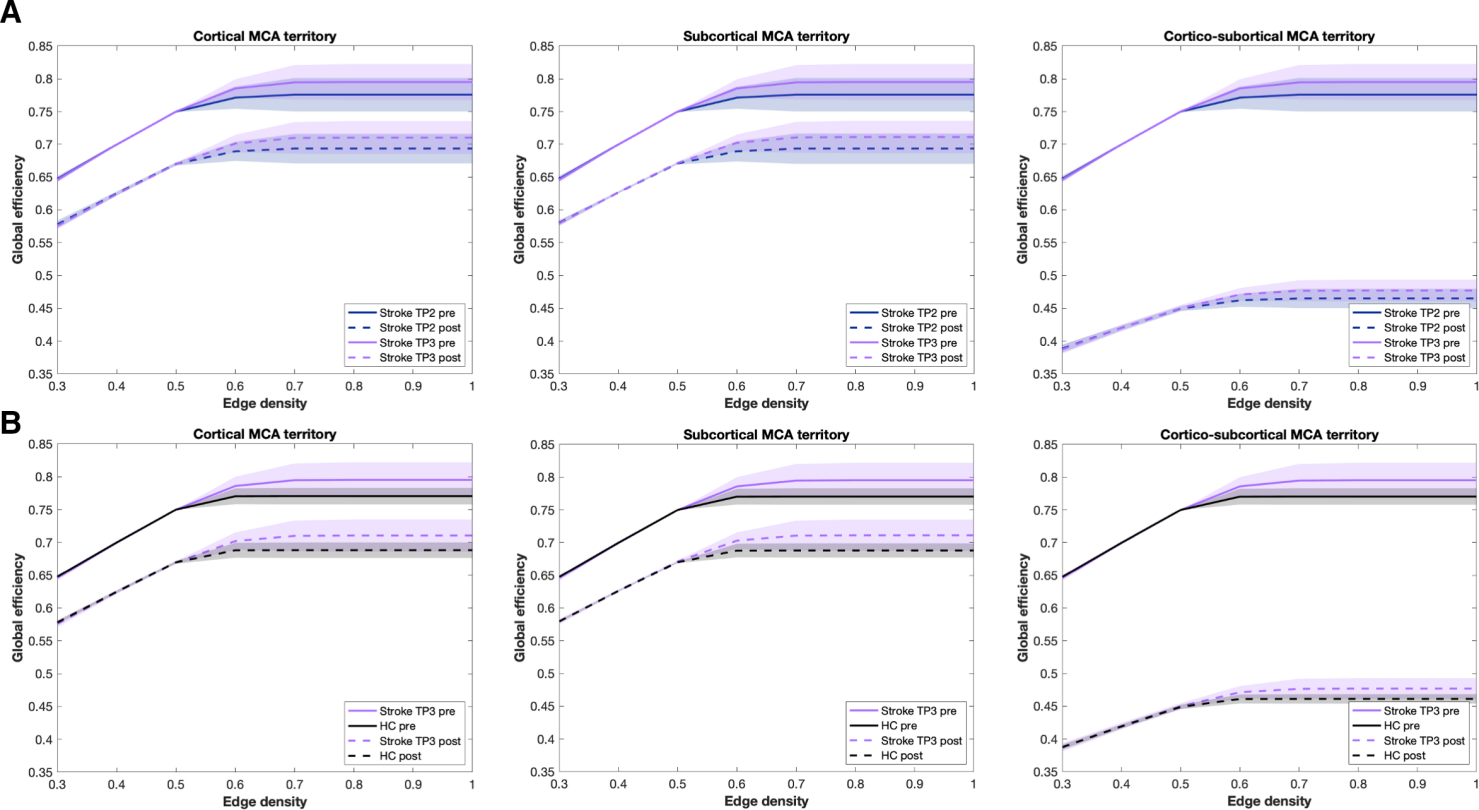
**Network resilience after simulation of clinically representative lesions.** Global efficiency after cortical, subcortical, and cortico-subcortical simulation of middle cerebral artery (MCA) strokes. **A**, Prelesion and postlesion comparison in patients at time points ( TP) 2 and TP3. **B**, Cross-sectional comparison between healthy controls (HC) and stroke patients at TP3, illustrated with mean global efficiency before (straight lines) and after attack (dotted lines), as well as SDs (filled areas) for each group.

(Figure 6) E_glob_ varied significantly over time (mixed model, F=18.97, *P*<0.001) and for cortico-subcortical attacks (mixed model, F=4716, *P*<0.001). There was no significant change in global efficiency from TP1 to TP2 after all 3 attack types (cortical: *P*_BH_=0.934; subcortical: *P*_BH_=0.916; cortico-subcortical: *P*_BH_=0.788). At TP3 compared to TP2 however, a higher resilience was observed after all types of attacks (cortical: *P*_BH_=0.045; subcortical: *P*_BH_=0.045; cortico-subcortical: *P*_BH_=0.047). When patients were compared to controls, a higher resilience was only found at TP3 (cortical: *P*_BH_=0.012; subcortical: *P*_BH_ =0.009; cortico-subcortical p_BH_ =0.009) but not at TP1 (cortical: *P*_BH_=0.261; subcortical: *P*_BH_=0.261; cortico-subcortical: *P*_BH_=0.245) nor at TP2 (*P*_BH_=0.261 in all cases).

#### Correlation Between Wiring Cost and Network Resilience

Increase in resilience between 3 weeks (TP2) and 3 months (TP3) after stroke was significantly correlated with the increase in wiring cost of the network (Spearman ρ=0.785; *P*=0.001; Figure S2).

## Discussion

In our population of highly selected patients with focal cortical strokes restricted to the primary motor cortex, we showed that resilience in brain networks increased between 3 weeks and 3 months after stroke. This was demonstrated using attacks mimicking clinically representative strokes by targeting specific or random nodes in the whole-brain network. This work represents the first evidence that network reorganization may prevent the consequences of a second stroke, at the price, however, of a higher wiring cost.

Resilience to attacks against the network increased at 3 months compared to 3 weeks, whereas patients had recovered completely from the first event. This was determined by a higher global efficiency (E_glob_) following all types of lesion simulation. Although network efficiency has not been studied in patients after a second lesion, previous observational studies have revealed distinct patterns of E_glob_ changes during recovery after a first stroke. In a study including patients with mainly subcortical lesions, a progressive decrease in E_glob_ in the contralateral hemisphere was observed.^27^ When E_glob_ was considered after normalization by random graphs, no changes were described up to 6 months after cortical or subcortical stroke.^28,29^ More in line with our results, a higher E_glob_ was described within the contralateral hemisphere in mice recovering well from intraluminal occlusion of the right MCA.^30^ Comparison of E_glob_ across studies proved, however, to be challenging, due to differences in patient population and in the methods applied to determine connectivity (structural versus functional). Importantly, in our study, the increase in E_glob_ was obtained at a higher network cost, estimated by mean node degree. The price of shorter paths and more efficient information propagation during stroke recovery could therefore be related to the development of new connections.

We interpret the higher E_glob_ following lesion stimulation at 3 months as the reflect of a higher capacity of the network to preserve information integration across the entire network in the case of a second event. Because clinical testing is not possible after lesion simulation, the clinical relevance of these findings remains hypothetical. However, animal studies demonstrated that longer delays between a first and second stroke were associated with a lower behavioral impact of the recurrent stroke.^2^ Our study suggests that new forms of network organization may increase brain resilience to new attacks. If de novo creation of new connections seems unlikely, we hypothesize that an increase in resilience may rather reflect the recruitment of preexisting connections that were not used in healthy controls for normal functioning. Our data suggest, at least in our patient population, that functional network reorganization after stroke does not exclusively aim at restoring initial reorganization but also tends towards developing a more resilient state to reduce the functional impact of further insults. We may also consider that mechanisms of regeneration participate in the network reorganization.^31–33^ Further studies investigating structural data such as cortical thickness, voxel-based morphometry, or structural connectivity may provide important information regarding the underlying mechanisms of resilience.

Interestingly, resilience was increased for all types of simulated lesions (subcortical, cortical, and cortico-subcortical). We hypothesized that widespread reorganization occurs because the location and size of the second lesion are not predictable and cannot be anticipated. However, it could also be speculated that resilience may differ according to stroke cause. For instance, resilience after a lacunar stroke would possibly develop in a configuration that would specifically protect from another lacunar stroke. Similarly, in a patient with a unilateral carotid stenosis, resilience may specifically aim at limiting the consequence of an ipsilateral stroke. In this case, resilience of brain networks may be part of a more global concept, including mechanisms related to hemodynamic impairment such as vasodilatation or development of collaterals.

This work purposefully focused on patients with small primary motor cortex lesions, with complete and early functional recovery. At 3 weeks, our patients had recovered their hand function. In patients with a larger index lesion or a lesion affecting a network hub, a greater decrease in E_glob_ could be expected early after stroke.^34^ We can nevertheless postulate that resilience could increase over time compared to earlier TPs. If patients would keep on improving their motor function beyond 3 weeks, mechanisms of resilience and recovery would coexist and become difficult to individualize. One of the advantages of our study population is the homogeneity of the lesions in terms of size and location. Furthermore, because patients have fully recovered clinically at 3 weeks, we were able to make the assumption that changes in connectivity occurring at later TPs were related to resilience and not solely to motor recovery.^15,35–38^ Nevertheless, generalizability of our findings to all types of stroke is challenging given the variability of stroke location, size, and clinical impact. Further studies are needed to determine whether similar changes occur with all types of lesions.

Methodologically, this study opens new perspectives for the study of network reorganization and resilience in stroke and other diseases. One experimental lesion simulation approach combines MRI-guided brain stimulation with functional connectivity MRI and high-density electroencephalography. In recent years, brain stimulation, when guided with MRI, has dramatically increased its spatial precision and high-density electroencephalography provides a detailed measure of brain physiology.^39,40^ If this strategy represents one of the most promising techniques to study brain resilience, it does not allow to mimic lesions that precisely correspond to those commonly observed in acute stroke patients. The use of node deletion to simulate lesions combines several essential characteristics for the study of brain resilience in human. First, it is a highly controllable and precise intervention that is fully noninvasive. To date, studies using node deletion to simulate lesions have been used to test the architecture of healthy or pathological networks with no intention to mimic clinically representative strokes..^9,20,41–45^ Here, we simulated clinically representative lesions by deleting contiguous nodes using masks of lesions observed in patients admitted to our stroke center. MCA lesions of various sizes and locations were considered in this proof-of-concept study. Large cortico-subcortical MCA lesions had a more dramatic effect on efficiency than lesions limited to the deep perforator of the MCA (subcortical lesion) or restricted to a superficial branch of the MCA (cortical lesion). This difference could be explained by both the size of the lesion and the importance of the nodes (hubs) affected by the different lesions.

Lesion simulation is an innovative approach, which may have major implications for stroke therapy. If the results of the current study can be confirmed in patients with a more heterogeneous pattern of stroke size and location, individualized neuromodulation strategies could be developed using transcranial magnetic stimulation or transcranial direct current stimulation to not only improve clinical function but also promote resilient network reconfigurations to limit the consequences of future attacks. This approach could also have important implications beyond the stroke field to support the development of individualized therapies for instance in neurodegenerative diseases, such as Alzheimer disease. Identification and promotion of network configurations that are more resilient to the degenerative process may have a huge clinical impact.^46^

There are several limitations to this study. First, we included only patients with discrete lesions limited to the primary motor cortex, who fully recovered at 3 weeks. The inclusion of highly selected patients limits the generalization of our results to other stroke patterns. The increased network resilience following stroke observed in our study may be limited to the setting of small lesions of the primary motor cortex and patients with swift and full recovery. Our results might be affected by the highly interconnected nature of the primary motor cortex.^47^ In patients with larger lesions or affecting hubs, a more severe impact on brain network topology could be initially expected early after stroke.^48^ This may concern lesions in regions previously identified as a rich club; for instance, the precuneus, superior frontal cortex, superior parietal cortex, hippocampus, putamen, thalamus.^49^ However, the greater impact that strokes have on E_glob_ in the acute phase does not exclude further increase of resilience over time. Finally, we limited our simulation to the deletion of nodes; therefore, structural connectivity was not taken into account. As real stroke lesions also affect white matter fiber tracts, the final impact of a second lesion could be more severe than reported here.^34^ Further studies should investigate these hypotheses.

## Conclusions

In the setting of a highly selected patient cohort limited to small strokes to the primary motor cortex, our results suggest that the optimal network reconfiguration following stroke may not be identical to prestroke architecture. Natural selection may have increased the robustness of neural networks by favoring their adaptability to unforeseen events.^50,51^ If confirmed in a larger stroke population with lesions of various sizes and locations, the results of our study may be relevant to inform neuromodulation strategies that intend to reconfigure network architecture during stroke recovery to also promote resilience.

## Article Information

### Sources of Funding

This work was supported by the Swiss National Science Foundation (320030_166535), the Privat Kredit Bank (PKB) Foundation, and the de Reuter Foundation.

### Disclosures

None.

### Supplemental Material

Tables S1–S3

Figures S1–S3

Supplemental Methods

## Supplementary Material


