# Supplementary material for: Preparing for a Second Attack: A Lesion Simulation Study on Network Resilience After Stroke
Source: Stroke. 2022 May 9;53(6):2038–47. doi: 10.1161/STROKEAHA.121.037372 (PMC10292575; doi:10.1161/STROKEAHA.121.037372)
Supplement: Supplementary file 1 [file str-53-2038-s001.pdf]

STROBE statement: Reporting guidelines checklist for cohort, case-control and cross-sectional studies

SECTION	ITEM NUMBER	CHECKLIST ITEM	REPORTED ON PAGE NUMBER:
**TITLE AND ABSTRACT**			
	1a	Indicate the study’s design with a commonly used term in the title or the abstract	1
	1b	Provide in the abstract an informative and balanced summary of what was done and what was found	4
**INTRODUCTION**			
Background and objectives	2	Explain the scientific background and rationale for the investigation being reported	4-5
	3	State specific objectives, including any pre-specified hypotheses	5
**METHODS**			
Study design	4	Present key elements of study design early in the paper	6
Setting	5	Describe the setting, locations, and relevant dates, including periods of recruitment, exposure, follow-up, and data collection	5
Participants	6a	Cohort study—Give the eligibility criteria, and the sources and methods of selection of participants. Describe methods of follow-up<br>Case-control study—Give the eligibility criteria, and the sources and methods of case ascertainment and control selection. Give the rationale for the choice of cases and controls<br>Cross-sectional study—Give the eligibility criteria, and the sources and methods of selection of participants	5
	6b	Cohort study—For matched studies, give matching criteria and number of exposed and unexposed<br>Case-control study—For matched studies, give matching criteria and the number of controls per case<br>Variables	NA
Variables	7	Clearly define all outcomes, exposures, predictors, potential confounders, and effect modifiers. Give diagnostic criteria, if applicable	6
Data sources/measurements	8*	For each variable of interest, give sources of data and details of methods of assessment (measurement). Describe comparability of assessment methods if there is more than one group.	5-6

SECTION	ITEM NUMBER	CHECKLIST ITEM	REPORTED ON PAGE NUMBER:
Bias	9	Describe any efforts to address potential sources of bias.	5
Study size	10	Explain how the study size was arrived at	5
Quantitative variables	11	Explain how quantitative variables were handled in the analyses. If applicable, describe which groupings were chosen and why .	7
Statistical methods	12a	Describe all statistical methods, including those used to control for confounding	7-10
	12b	Describe any methods used to examine subgroups and interactions	9
	12c	Explain how missing data were addressed	NA
	12d	Cohort study—If applicable, explain how loss to follow-up was addressed<br>Case-control study—If applicable, explain how matching of cases and controls was addressed<br>Cross-sectional study—If applicable, describe analytical methods taking account of sampling strategy	NA
	12e	Describe any sensitivity analyses	NA
**RESULTS**			
Participants	13a	Report numbers of individuals at each stage of study—eg numbers potentially eligible, examined for eligibility, confirmed eligible, included in the study, completing follow-up, and analysed	5
	13b	Give reasons for non-participation at each stage	5
	13c	Consider use of a flow diagram	NA
Descriptive Data	14a	Give characteristics of study participants (eg demographic, clinical, social) and information on exposures and potential confounders	**5**
	14b	Indicate number of participants with missing data for each variable of interest	5
	14c	Cohort study—Summarise follow-up time (eg, average and total amount)	10
Outcome Data	15*	Cohort study—Report numbers of outcome events or summary measures over time<br>Case-control study—Report numbers in each exposure category, or summary measures of exposure<br>Cross-sectional study—Report numbers of outcome events or summary measures	10
Main Results	16a	Give unadjusted estimates and, if applicable, confounder-adjusted estimates and their precision (e.g. 95% confidence interval). Make clear which confounders were adjusted for and why they were included	10-11

SECTION	ITEM NUMBER	CHECKLIST ITEM	REPORTED ON PAGE NUMBER:
	16b	Report category boundaries when continuous variables were categorized	10-11
	16c	If relevant, consider translating estimates of relative risk into absolute risk for a meaningful time period	NA
	16d	Report results of any adjustments for multiple comparisons	10-11
Other Analyses	17a	Report other analyses done—e.g. analyses of subgroups and interactions, and sensitivity analyses	NA
	17b	If numerous genetic exposures (genetic variants) were examined, summarize results from all analyses undertaken	
	17c	If detailed results are available elsewhere, state how they can be accessed	
**DISCUSSION**			
Key Results	18	Summarise key results with reference to study objectives	11
Limitations	19	Discuss limitations of the study, taking into account sources of potential bias or imprecision. Discuss both direction and magnitude of any potential bias	13
Interpretation	20	Give a cautious overall interpretation of results considering objectives, limitations, multiplicity of analyses, results from similar studies, and other relevant evidence	11-13
Generalisability	21	Discuss the generalisability (external validity) of the study results<br>Other information	13
**FUNDING**			
	22	Give the source of funding and the role of the funders for the present study and, if applicable, for the original study on which the present article is based	13
