# Supplementary material for: Preparing for a Second Attack: A Lesion Simulation Study on Network Resilience After Stroke
Source: Stroke. 2022 May 9;53(6):2038–47. doi: 10.1161/STROKEAHA.121.037372 (PMC10292575; doi:10.1161/STROKEAHA.121.037372)
Supplement: Supplementary file 2 [file str-53-2038-s002.pdf]

SUPPLEMENTAL MATERIAL.

van Assche M, Klug J, Dirren E, Richiardi J, Carrera E. Preparing for a second attack: a lesion simulation study on network resilience after stroke.

I. Patient characteristics

II. Spontaneous changes in degree and edge count

III. Effect of initial lesion characteristics on spontaneous changes of global efficiency

IV. Theoretical limits of network resilience

V. Brainnetome regions

I. Patient characteristics

Table S1. Patient characteristics and initial stroke lesion characteristics. Male (F); Female (F); Left (L); Right (R).

Patient	Gender	Age (years)	Lesion side	Lesion volume (cm^3)
p1	M	60	L	2.43
p2	F	52	R	0.49
p3	M	58	L	0.13
p4	M	79	L	0.46
p5	F	81	R	4.69
p6	F	87	L	1.5
p7	M	86	L	0.52
p8	M	78	R	1.73
p9	F	73	L	0.1
p10	M	81	L	0.29
p11	F	88	L	1.69
p12	M	60	L	0.29
p13	F	64	R	0.62
p14	M	83	L	0.48
p15	M	76	L	0.71
p16	M	61	L	0.69

II. Spontaneous changes in degree and edge count

Degree, mean degree and edge count were measured at each of the three time points in patients (10 days, 3 weeks, 3 months) and in controls. To account for network density, the area under the curve (AUC) over the selected density range ([0.3-1.0]) was derived for degree, mean degree and edge count. We used a linear mixed model with respectively AUC(mean degree) and AUC(edge count) as the dependent variables, "timepoint", "lesion side" and "lesion volume" as fixed effects and "subjects" as a random effect. At timepoint 3, we observe a significantly higher number of edges (mixed effects model, F=4.363, p=0.023) which is reflected in a significantly higher mean degree (mixed effects model, F= 5.406, p=0.028).

Figure S1. Changes in network mean degree over time. Boxplots for AUCs of mean degree in patients (ST, shown in yellow) and healthy controls (HC, shown in green) at each timepoint. Each box extends from the 25th percentile to the 75th percentile with a line indicating the median. Upper and lower whiskers show the range up to the upper and lower extremes (+/- 1.5*inter-quartile range). Outliers are represented by grey diamond shapes.

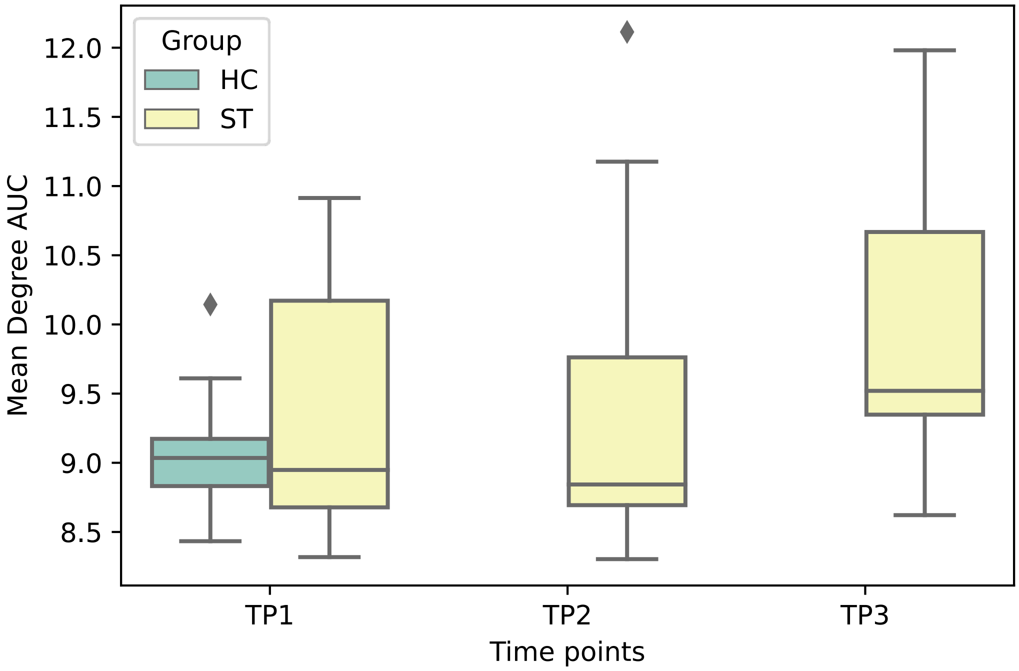

We further evaluated the change in degree for each region of interest (ROI) of the Brainnetome atlas between timepoint 1 and 3 in subjects. This was defined as the mean of differences of

AUC(degree) over all subjects at each ROI. Table S2 displays the 10 ROIs with the biggest gain in degree between timepoints 1 and 3.

Anecdotally we observe that the greatest change in degree pertains to non-motor networks. Furthermore, we note the high representation of subcortical nuclei of which the putamen, hippocampus and thalamus are part of the so-called “rich club”, a selection of network hubs, which are not only highly connected to the rest of the brain but are also highly connected between each other[45].

Figure S2. Correlation of change in global efficiency and mean degree between timepoint 2 and 3. Individual values are represented by blue dots. A regression line (straight blue line) is plotted with 95% confidence intervals (shaded area). Distributions for both variables are shown as histograms.

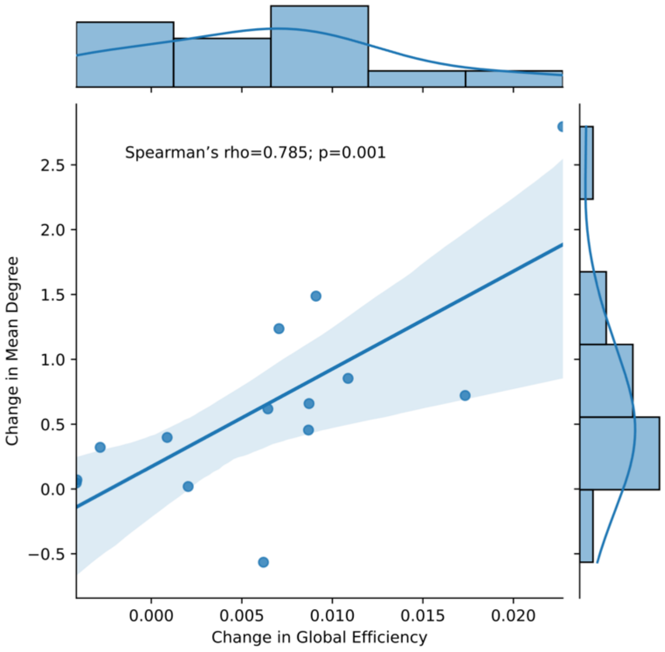

Table S2. Top 10 regions of interest by gain in degree. Regions of interest are displayed as anatomical Brainnetome region and modified Cyto-architectonic descriptions. Ipsilesional (I); Contralesional (R).

Region of interest	Hemisphere
A40rv, rostroventral area 40 (PFop)	I
vmPu, ventromedial putamen	C
rTtha, rostral temporal thalamus	C
dIa, dorsal agranular insula	I
cpSTS, caudoposterior superior temporal sulcus	C
A12/47l, lateral area 12/47	I
vmPu, ventromedial putamen	I
vIa, ventral agranular insula	C
aSTS, anterior superior temporal sulcus	C
cHipp, caudal hippocampus	C

III. Effect of initial lesion side and volume on spontaneous changes of global efficiency

To assess the effect of the volume and laterality of the initial stroke lesion on changes of global efficiency (E_{glob}) over time we used a linear mixed model with E_{glob} as the dependent variable, "lesion volume", "lesion side" and "timepoint" as fixed effect factors and "subjects" as a random effect. Median initial stroke lesion volume was 0.57 cm^3. Differences in E_{glob} were not statistically significant for different initial lesion volumes (mixed model, $F= 1.541$; $p= 0.235$) or lesion side (mixed model, $F= 0.222$, $p= 0.645$).

IV. Theoretical limits of network resilience

To assess the theoretical limits of the increase in resilience observed after stroke, we performed repeated random attacks on controls and patients at timepoint 3. We sequentially deleted random nodes and compared global efficiency between patients and controls after every node deletion. This process was repeated 100 times. T-tests with false discovery rate (FDR) corrections (Benjamini–Hochberg procedure) for multiple comparisons were used[17].

As expected, the difference in global efficiency after attack converges to zero with increasing number of nodes attacked. Concurrently, the difference in resilience between controls and patients at timepoint 3 becomes increasingly non-significant.

It is hard to draw clinical conclusions from these simulated lesions as no clinical testing is available. Nonetheless, it is likely that the gain in resilience after a first stroke in increasingly offset as the size of the second lesion approaches the size of a hemisphere (120 nodes).

Figure S3. Difference in global efficiency after repeated sequential random attack between patients at timepoint 3 and controls. Scatterplot of the difference in mean global efficiency between patients at timepoint 3 and controls after sequential random attack (**A**). Scatterplot of FDR corrected p-values for the difference in global efficiency between patients at timepoint 3 and controls after sequential random attack (**B**). The sequential random deletion of nodes was repeated for 100 iterations. A color scale was applied for visual adjustment of point density with number of deleted nodes, ranging from light red (no deleted node) to blue (all nodes deleted).

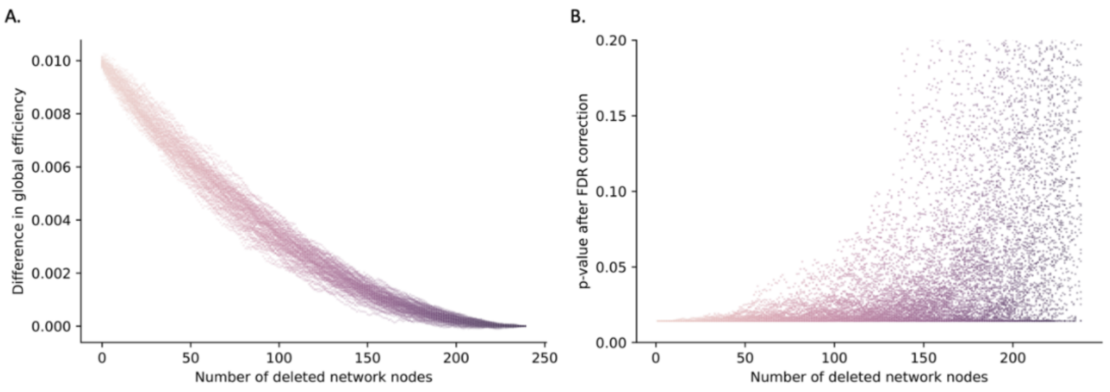

V. Brainnetome regions

Table S3. Brainnetome nomenclature for clinically representative lesions.

Brainnetome abbreviations for regions that are part of the clinically representative lesions used in this work are displayed with corresponding anatomical lobe, gyrus and a modified cyto-architectonic description, as well as the type of lesion of which they are part. This table is adapted from Table 1 of the following paper[17].

Fan L, Li H, Zhuo J, Zhang Y, Wang J, Chen L, Yang Z, Chu C, Xie S, Laird AR, et al. The Human Brainnetome Atlas: A New Brain Atlas Based on Connectional Architecture. *Cereb Cortex*. 2016;26:3508-3526.

**Lobe**	**Gyrus**	**Abbreviation**	**Description**	**Lesion type**
**Frontal Lobe**	Superior Frontal Gyrus	A8m	medial area 8	cortico-subcortical
		A8dl	dorsolateral area 8	cortico-subcortical
		A9l	lateral area 9	cortico-subcortical
		A6dl	dorsolateral area 6	cortico-subcortical
		A9m	medial area 9	cortico-subcortical
	Middle Frontal Gyrus	A9/46d	dorsal area 9/46	cortico-subcortical
		IFJ	inferior frontal junction	cortico-subcortical
		A9/46v	ventral area 9/46	cortico-subcortical
		A8vl	ventrolateral area 8	cortico-subcortical
		A6vl	ventrolateral area 6	cortico-subcortical

		A10l	lateral area10	cortico-subcortical
	Inferior Frontal Gyrus	A44d	dorsal area 44	cortico-subcortical
		IFS	inferior frontal sulcus	cortico-subcortical
		A45c	caudal area 45	cortico-subcortical
		A45r	rostral area 45	cortico-subcortical
		A44op	opercular area 44	cortico-subcortical
		A44v	ventral area 44	cortico-subcortical
	Orbital Gyrus	A12/47o	orbital area 12/47	cortico-subcortical
		A11l	lateral area 11	cortico-subcortical
		A13	area 13	cortico-subcortical
		A12/47l	lateral area 12/47	cortico-subcortical
	Precentral Gyrus	A4hf	area 4 (head and face region)	cortical, cortico-subcortical
		A6cdl	caudal dorsolateral area 6	cortico-subcortical
		A4ul	area 4 (upper limb region)	cortico-subcortical
		A4tl	area 4 (tongue and larynx region)	cortical, cortico-subcortical
		A6cvl	caudal ventrolateral area 6	cortico-subcortical

**Temporal Lobe**	Superior Temporal Gyrus	A41/42	area 41/42	cortical, cortico-subcortical
		TE1.0 and TE1.2	TE1.0 and TE1.2	cortical, cortico-subcortical
		A22c	caudal area 22	cortical, cortico-subcortical
		A38l	lateral area 38	cortico-subcortical
		A22r	rostral area 22	cortical, cortico-subcortical
		aSTS	anterior superior temporal sulcus	cortico-subcortical
**Parietal Lobe**	Inferior Parietal Lobule	A40rd	rostrodorsal area 40	cortical, cortico-subcortical
		A40rv	rostroventral area 40	cortical, cortico-subcortical
	Postcentral Gyrus	A1/2/3ulhf	area 1/2/3 (upper limb, head, and face region)	cortical, cortico-subcortical
		A1/2/3tonIa	area 1/2/3 (tongue and larynx region)	cortical, cortico-subcortical
		A2	area 2	cortical, cortico-subcortical
**Insular Lobe**	Insular Gyrus	G	hypergranular insula	cortical, cortico-subcortical

		vIa	ventral agranular insula	cortico-subcortical
		dIa	dorsal agranular insula	cortico-subcortical
		dIg	dorsal granular insula	cortical, cortico-subcortical
		dId	dorsal dysgranular insula	cortico-subcortical
**Subcortical Nuclei**	Amygdala	mAmyg	medial amygdala	sub-cortical
		lAmyg	lateral amygdala	sub-cortical, cortico-subcortical
	Basal Ganglia	vCa	ventral caudate	sub-cortical, cortico-subcortical
		GP	globus pallidus	sub-cortical, cortico-subcortical
		NAC	nucleus accumbens	sub-cortical, cortico-subcortical
		vmPu	ventromedial putamen	sub-cortical, cortico-subcortical
		dCa	dorsal caudate	sub-cortical, cortico-subcortical
		dlPu	dorsolateral putamen	sub-cortical, cortico-subcortical
	Thalamus	mPMtha	pre-motor thalamus	sub-cortical, cortico-subcortical

		Stha	sensory thalamus	sub-cortical, cortico-subcortical
		PPtha	posterior parietal thalamus	sub-cortical, cortico-subcortical
		cTtha	caudal temporal thalamus	sub-cortical, cortico-subcortical
		lPFtha	lateral pre-frontal thalamus	sub-cortical, cortico-subcortical
